# Effectiveness of a high-intensity laser for improving hemiplegic shoulder dysfunction: a randomized controlled trial

**DOI:** 10.1038/s41598-024-57453-9

**Published:** 2024-03-28

**Authors:** Nadia Mohamed Abdelhakiem, Marwa Shafiek Mustafa Saleh, Magdy M. A. Shabana, Hisham A. Abd EL Wahaab, Haitham M. Saleh

**Affiliations:** 1 Department of Physical Therapy for Neuromuscular Disorders and Surgery, Faculty of Physical Therapy, Deraya University, Minya, Egypt; 2 Basic Science Department, Faculty of Physical Therapy, Cairo University, Cairo, Egypt; 3 Department of Physical Therapy, Faculty of Applied Medical Sciences, Al‐Zaytoonah University of Jordan, Amman, Jordan; 4 Department of Musculoskeletal Disorders, Faculty of Physical Therapy, Deraya University, Minya, Egypt; 5 Cairo University Hospital, Cairo University, Cairo, Egypt; 6 Department of Physical Therapy for Internal Medicine, Chest and Cardiology, Faculty of Physical Therapy, Deraya University, Minya, Egypt; 7 Department of Physical Therapy for Basic Science Faculty of Physical Therapy, Deraya University, Minya, Egypt

**Keywords:** Neuroscience, Neurology

## Abstract

Hemiplegic shoulder pain (HSP) is a common complication that occurs after stroke and has been reported in up to 84% of hemiplegic patients. One of the recommended treatment options for shoulder pain is high-intensity laser therapy (HILT). This study aimed to determine the effectiveness of high-intensity laser therapy on pain, function and hand grip strength in patients with hemiplegic shoulder dysfunction. Forty-four hemiplegic patients were randomly divided into two groups: Group 1 (study group, n = 22) received 3 HILT sessions a week for three weeks in combination with three sessions of therapeutic exercise per week for three weeks, and Group 2 (control group, n = 22) received a conventional exercise program for HSP three times a week for three weeks. Shoulder pain was evaluated using the McGill pain questionnaire (MPQ), the functional outcome of the shoulder was evaluated with the University of California–Los Angeles functional scale (UCLA), and handgrip strength was evaluated with a hydraulic hand dynamometer. The increase in the UCLA scores and the decrease in the MPQ scores after treatment were significant in the study group (p < 0.001) as well as in the control group (p < 0.05) in comparison with the pretreatment between-group comparison. Additionally, the increase in hand grip strength was significant in both groups after treatment (p < 0.001). The study group showed significant improvement over the control group with respect to the UCLA score, handgrip strength, and MPQ score (p < 0.001). HILT combined with therapeutic exercise provides greater improvement than therapeutic exercise alone in terms of hemiplegic shoulder pain, dysfunction, and handgrip strength.

## Introduction

In hemiplegic shoulders, 3 related types of impairment are observed: pain, static or structural disorders, and dynamic or functional disorders. All three factors must be considered in concert to investigate shoulder complications comprehensively^1^.

Hemiplegic shoulder pain (HSP), which is a frequent and debilitating complication after a stroke, can affect quality of life^2^. Hemiplegic shoulder pain is often used to describe a collection of complex problems and diagnoses. However, its causes have not been identified^3^.

HSP mostly occurs two to three months after a stroke^4^. Prevalence rates vary between 22 and 47%, while incidence rates vary between 10 and 22%^5^. HSP seems to decrease shoulder function at 12 months after acute stroke. This is very important because HSP is a potentially preventable (or reversible) factor^6^.

Ultrasonography enables the identification of common shoulder pathologies after stroke. The most prevalent pathology in hemiplegic shoulders pertained to the long head of the biceps tendon (41.4%), followed by the supraspinatus tendon (33.2%), subdeltoid bursa (29.3%), acromioclavicular joint (15.0%), and subscapularis tendon (9.2%). The common pathological findings include bicipital peri-tendinous effusion (39.2%), bicipital tendinopathy (35.5%), and supraspinatus tendinopathy (24.6%). Biceps long head tendon and supraspinatus tendon abnormalities are significantly more common in the hemiplegic (vs. contralateral) shoulders^7^.

Ninety percent of patients affected by stroke have upper extremity impairment^8^. The limitations of movement range from permanently affected upper extremities, leading to difficulty in normal life for poststroke patients^9^ and difficulty performing activities of daily living due to hemiplegia^10^.

There are several causes of HSP resulting from stroke. Examples include muscle flaccidity surrounding the shoulder joint, shoulder subluxation, shoulder-hand syndrome, exaggerated muscle tone, subacromial impingement syndrome, adhesive capsulitis, brachial plexus lesion, and thalamic syndrome^11,12^.

Muscle plasticity around the shoulder joint is one of the most common causes of shoulder pain in hemiplegic shoulder patients. Intramuscular injection of botulinum toxin (BoNT) and suprascapular nerve blocks (SSNB) are superior to a placebo, with SSNB having the highest probability (53.3%) and appearing to be the best treatment in the fourth week, followed by intramuscular BoNT injections (42.6%). Intramuscular BoNT injections were better than placebo injections between the 4th and 24th weeks. Intramuscular BoNT injections had the highest probability (79.8%) of being the best treatment between the 4th and 24th weeks^13^.

Stroke is usually associated with muscle weakness or paresis, muscle tone abnormalities, and proprioceptive damage, which may leave the shoulder complex unstable and consequently malaligned^14^.

One of the recommended treatment options for shoulder pain is high-intensity laser therapy (HILT)^15^. Because of its photomechanical, thermal, electrical, and biostimulating effects on deep tissues, HILT has traditionally been used to treat conditions that low-intensity laser therapy (LILT) cannot, such as increased microcirculation, accelerated tissue regeneration, decreased swelling, inflammation, and pain^16^.

Compared to LILT, HILT provides benefits such as increased power, deeper tissue penetration, shorter emission duration, and longer rest periods that prevent heat accumulation^17^.

Subacromial impingement syndrome, rotator cuff tendinopathy, and adhesive capsulitis have all been proven to respond effectively with HILT in the majority of recent studies^15^.

Recently, studies have demonstrated that HILT is useful for treating a variety of shoulder impairments, including subacromial impingement syndrome, rotator cuff tendinopathy, and adhesive capsulitis. To the best of our knowledge, the effects of HILT on pain, shoulder function, and hand grip strength in individuals with HSP are still unclear.

The aim of this study was to investigate the effect of HILT in addition to conventional physical therapy on hemiplegic shoulder pain, dysfunction and handgrip strength.

### Hypothesis

The addition of a high-intensity laser to conventional physical therapy has no significant effect on improving hemiplegic shoulder pain, dysfunction or handgrip strength.

## Materials and methods

This prospective randomized controlled trial was conducted with approval from the ethics committees of the faculty of physical therapy at Cairo University (approval NO: P.T. REC/012/004877, registration on clinical trials.gov, identifier: NCT05595720, date of first registration: 27/10/2022). All methods were performed according to the relevant guidelines and regulations.

### Inclusion criteria

HSP patients aged 40–75 years^18^ with unilateral hemiplegia for the first time and a hemiplegia duration of 6 months.

Patients with a history of inflammation-related rheumatic disease, cervical radiculopathy, diabetes, cardiovascular disease, cardiac pacemakers, shoulder operations, or shoulder injection during the previous three months were excluded.

### Sample size

Sample size calculation was performed using G*POWER statistical software (version 3.1.9.2; Universitat Kiel, Germany). The UCLA score was derived from a pilot study conducted on five subjects in each group, and the results revealed that the required size of each group was 22 subjects. The calculations were made using α = 0.05, a power of 80% and an effect size of 0.88.

### Study design

**Figure 1:**
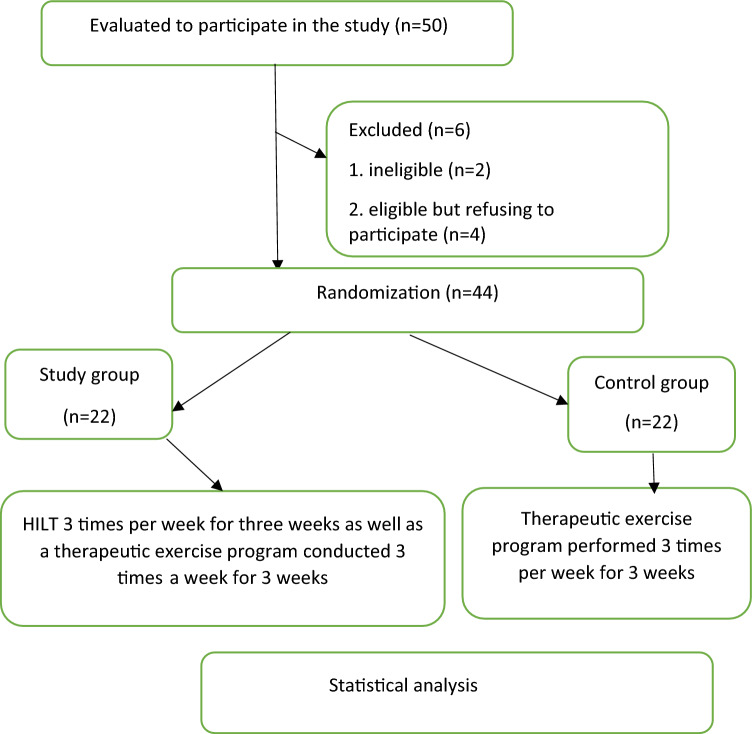
Flow chart.

In accordance with the sample size calculation by G. Power, forty-four patients were selected from a total of 50 subjects with hemiplegic shoulder dysfunction. The forty-four patients were selected from the outpatient clinic of Deraya University in Elmenia by a neurologist, fulfilled the inclusion criteria, agreed to participate in the study by signing a consent form, and were randomized into 2 groups utilizing the closed envelope method (Fig. 1). Informed consent from all subjects was obtained—for both study participation and publication of identifying information/images in an online open-access publication.

After a patient agreed to be involved in the study, the randomization was performed as follows: patients who had odd numbers were enrolled in the study group, and patients with even numbers were enrolled in the control group.

Patient pain was assessed by the McGill pain questionnaire (MPQ), the functional outcome of the shoulder was assessed by the University of California Los Angeles functional scale (UCLA), and handgrip strength was assessed by a hydraulic hand dynamometer.

The McGill Pain Questionnaire was employed to assess pain. The Pain Rating Index and a 5-point pain intensity scale (representing pain intensity) are derived from responses to the scale's four subscales that assess sensory, affective, evaluative, and other elements of pain. Items counted: The Pain Rating Index's 78 pain description items are divided into 20 subclasses, each of which consists of 2–6 words belonging to one of four primary subscales: sensory (subclasses from 1 to 10), emotional (subclasses from 11 to 15), evaluative (subclass 16), or miscellaneous (subclasses from 17 to 20). An additional one-point pain scale is included^19,20^.

The patient's activity levels were evaluated using the UCLA activity score. A before-and-after measurement of this index is possible. Using answers to five questions about how often the patient uses his or her shoulders, we calculated the shoulder activity level score. Moreover, two questions examined individuals' participation in athletic sports^21^.

**Figure 2:**
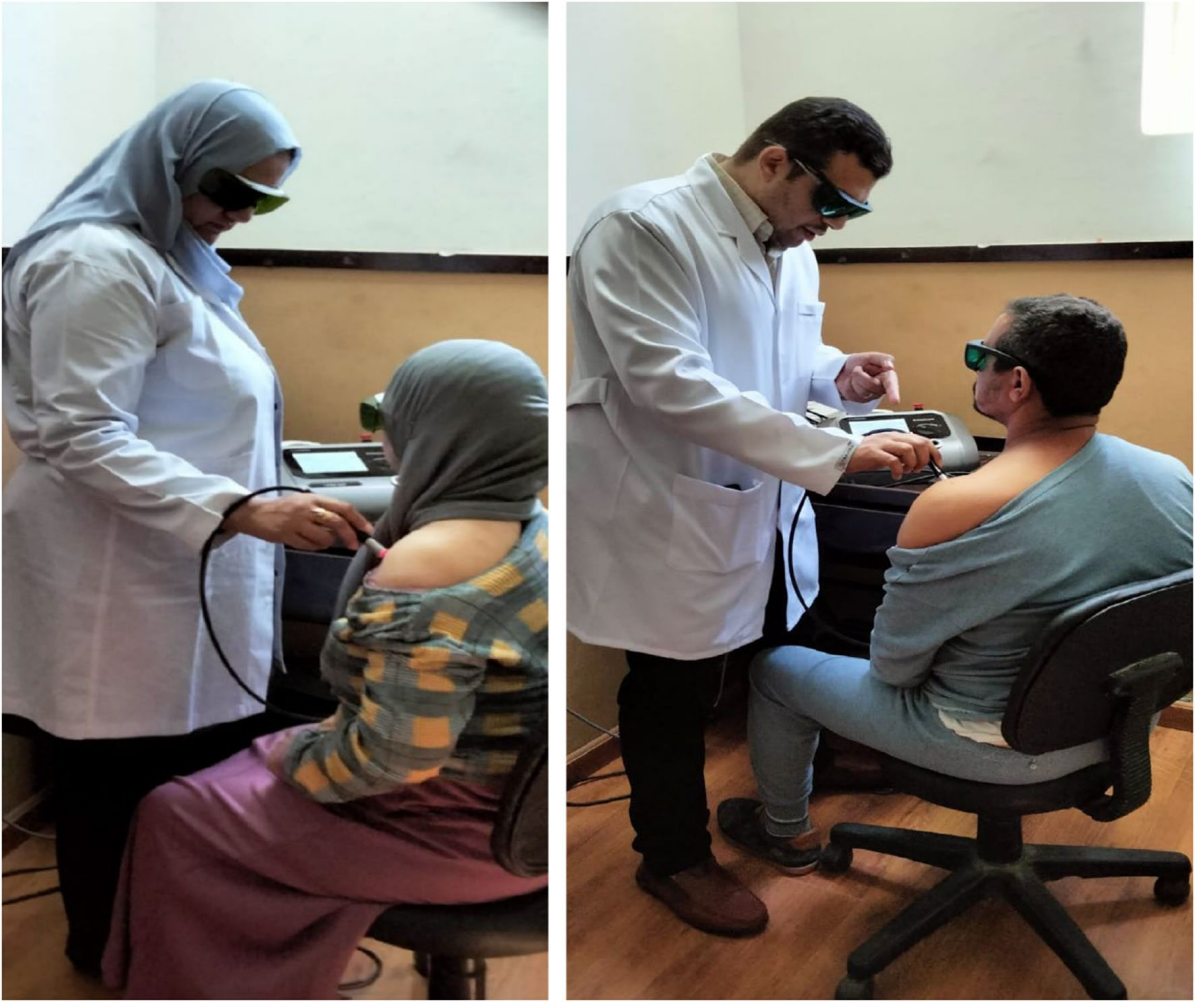
The application of high-intensity laser therapy (HILT) for the treatment of hemiplegic shoulder pain (HSP) in stroke patients.

Patients were divided into two main groups: Group 1 (study group, n = 22) was given HILT three times a week for three weeks, and a therapeutic exercise program was implemented three times a week for three consecutive weeks. Group 2 (control group, n = 22) was given a conventional exercise program for HSP three times a week for three consecutive weeks, and reassessment of the patients was performed one week after the last session of the planned treatment period. (Fig. 2).

Both groups were assessed by the MPQ score, UCLA shoulder score, and handgrip strength using a hydraulic hand dynamometer before and after treatment. The program consisted of ITO (LAZR-207) at a high intensity (25 Hz) with a frequency of 10 W and a dose of 12 j/cm2 for 2 min, which was applied on the same day^22^. The HILT group had two phases of device application to the shoulder area. Both phases I and II of the administration involved continuous circular motion. An analgesic effect was achieved in the first three sessions with a 75-s intermittent period at 8 W and 10 J/cm^2^ for an overall of 100 J. In the subsequent six sessions, the biostimulating impact lasted continuously for 30 s at a dose of 12 W 120 J/cm^2^. The laser treatment was administered perpendicular to the joint line, with the patient's arm in medial rotation at the back and in lateral rotation at the front. The glenohumeral joint was irradiated at eight different locations. Nine HILT sessions were administered over 3 weeks^23^.

The conventional exercise program for hemiplegic shoulder pain (HSP) was based on Kabat approaches a. Active abduction exercise with physical therapist assistance; b. Passive shoulder flexion with physiotherapist assistance; b Shoulder flexion/extension with active-resisted exercise^24^.

### Statistical analysis

Subject characteristics were compared across groups utilizing an unpaired t test. The sex, stroke type, dominant hand, and affected side were compared between groups utilizing the chi-squared test. To ensure that the data were normally distributed, we performed the Shapiro‒Wilk test. Group homogeneity of variance was analyzed using Levene’s test. To compare the effects on the UCLA score, MPQ score, and hand grip strength within and between groups, two-way mixed MANOVA was used. Multiple comparisons were performed through the use of Bonferroni post hoc correction. A p < 0.05 threshold was used for all the statistical analyses. All statistical analyses were performed using IBM SPSS Statistics for Windows, Version 22 (IBM SPSS, Chicago, IL, USA).

## Results

### Subject characteristics

The characteristics of the participants in both the study group and the control group are shown in Table 1. In terms of age, duration of pain, sex, stroke type, dominance and affected side, there were no statistically significant differences among the groups (p > 0.05).

**Table 1:** Comparison of subject characteristics between the study and control groups:

	Study group	Control group	Statistics	p value
Age, mean ± SD (years)	54.77 ± 7.89	55.63 ± 8.16	(t = -0.72)	0.47
BMI, mean ± SD (years)	28.09 ± 2.18	27.64 ± 2.13	(t = -0.70)	0.49
Duration of pain, mean ± SD (weeks)	5.00 ± 1.72	5.05 ± 1.78	(t = -0.09)	0.93
Sex, n (%)				
Female	10 (45.5%)	9 (40.9%)	(χ^2^ = 0.09)	0.76
Male	12 (54.5%)	13 (59.1%)		
Stroke type, n (%)				
Ischemic	18 (81.8%)	15 (68.2%)	(χ^2^ = 1.09)	0.48
Hemorrhagic	4 (18.2%)	7 (31.8%)		
Stroke onset, n (%)				
Gradual	18 (81.8%)	15 (68.2%)	(χ^2^ = 1.09)	0.48
Sudden	4 (18.2%)	7 (31.8%)		
Dominant side, n (%)				
Right	21 (95.5%)	22 (100%)	(χ^2^ = 1.02)	1
Left	1 (4.5%)	0 (0%)		
Affected side, n (%)				
Right	14 (63.6%)	12 (54.5%)	(χ^2^ = 0.37)	0.54
Left	8 (36.4%)	10 (45.5%)		

*SD* standard deviation, *t* unpaired t value, *χ*^*2*^ chi-squared value; p value, level of significance.

#### Impact of treatment on UCLA, MPQ scores, and hand strength

A significant interaction between treatment and time was found using two-way mixed MANOVA (F = 278.93, p < 0.001). The main effect of time was statistically significant (F = 815.04, p < 0.001). The main impact of treatment was statistically significant (p < 0.001).

The increase in UCLA and decrease in MPQ scores after treatment were significant in the study group (p < 0.001) and the control group (p < 0.05) compared with the pretreatment within-group comparison. Additionally, an increase in hand grip strength was significant in both groups after treatment (p < 0.001).

However, before treatment, there was no statistically significant difference (p > 0.05) between the groups. After receiving treatment, the UCLA, handgrip strength test, and MPQ improved significantly in the study group compared with the control group (p < 0.001). Post hoc power analysis revealed a power of 100% (Table 2).

**Table 2:** Mean UCLA and MPQ scores and hand strength before and after treatment in the study and control groups.

	Study group	Control group	MD (95% CI)	p value
	Mean ± SD	Mean ± SD		
UCLA score				
Pretreatment	9.68 ± 2.23	9.59 ± 1.86	0.09 (− 1.16:1.34)	0.88
Posttreatment	19.77 ± 3.03	10.31 ± 2.16	9.46 (7.84; 11.06)	0.001
MD (95% CI)	− 10.09 (− 10.72; − 9.46)	− 0.72 (− 1.35; − 0.09)		
	p = 0.001	p = 0.02		
MPQ scores				
Pretreatment	61.45 ± 6.5	59.86 ± 6.77	1.59 (− 2.44; 5.62)	0.43
Posttreatment	28 ± 6.98	57 ± 6.74	− 29 (− 33.17; − 24.82)	0.001
MD (95% CI)	33.45 (30.95; 35.95)	2.86 (0.36; 5.36)		
	p = 0.001	p = 0.02		
Hand grip strength (lb)				
Pretreatment	4.28 ± 0.94	4.63 ± 1.36	− 0.35 (− 1.06; 0.36)	0.33
Posttreatment	15.55 ± 1.54	11.86 ± 2.02	3.69 (2.58; 4.77)	0.001
MD (95% CI)	− 11.27 (− 12.05; − 10.48)	− 7.23 (− 8.01; − 6.45)		
	p = 0.001	p = 0.001		

Mean, *SD* standard deviation, *MD* mean difference, *CI* confidence interval.

p value, level of significance.

## Discussion

In the present study, both the clinical and control groups showed significant improvements in shoulder pain, disability, and hand grip strength directly after treatment compared with before treatment, with more favorable results for the study group. These improvements in pain and dysfunction may be attributed to the ability of HILT to reduce inflammation and relieve pain symptoms by improving cell metabolism, blood flow, and vascular permeability^25^. The improvement in shoulder pain enables the patient to improve the use of his or her hand, which results in increased handgrip strength.

Therefore, these results agree with those of Azra Karabegović et al., who reported that LASER therapy has an analgesic effect and reduces edema. Compared with electrotherapy (TENS, stable galvanization), this therapeutic modality significantly improved the pain experienced in the shoulder and hand of the hemiplegic arm and increased the range of motion in the shoulder of the affected arm^26^. Similarly, Kamal et al. reported that high-level LASER therapy produced greater increases in shoulder flexibility in impingement syndrome patients. HILT has shown a superior impact on reducing pain and enhancing mobility in short-term applications^27^. Ebid and El-Sodany reached the same conclusion that HILT has a greater effect and further long-term impact than a SHAM laser regarding pain decline and enhancing shoulder ROM^28^**.**

Pekyavas and Baltaci conducted an analysis of pretreatment and posttreatment outcomes and found that the addition of HILT to other physiotherapy interventions led to favorable results compared to those with placebo^15^. The results of the present study parallel those of a study published in 2015 by Sae Hoon et al., who assessed the clinical significance of HILT in patients with frozen shoulders^29^.

Akkurt et al. reported noticeable improvements in pain and hand grip strength after analysis of long-term HILT treatment effects^30^**.** Additionally, Kaydok et al. studied HILT and reported that it had a more significant effect on hand grip strength than LILT^31^.

The results of the current study are not congruent with the outcomes of a study published by Javier et al., who reported a treatment that continued for 3 consecutive weeks with a total of 15 sessions (5 sessions/week). In every session, patients with subacromial syndrome receiving HILT (experimental group) or sham-laser therapy (controlled group) reported that the HILT protocol combined with exercise (including stretching and strengthening exercises) did not improve pain or function better than exercise alone^32^.

Although several studies have demonstrated the therapeutic effectiveness of HILT for controlling musculoskeletal pain, Ezzati et al. determined that it is not yet time to reach such a conclusion. Indeed, it is possible that the therapeutic benefits of HILT could be enhanced by the inclusion of related cointerventions^33^.

## Conclusion

This study revealed that HILT accompanied by therapeutic exercises provides better improvement than therapeutic exercises alone in terms of hemiplegic shoulder pain, dysfunction, and hand grip strength.

## Limitations

There was no long-term follow-up of the patients in this study. Consequently, future studies are recommended to determine the long-term effect of high-power laser application on both hemiplegic shoulder pain and dysfunction.

## Recommendation

It is recommended to test laser treatment against sham or placebo therapy in future studies.

## Data Availability

The datasets used and analyzed during the current study are available from the corresponding author upon reasonable request.
